# Role of Serotonergic System on Modulation of Depressogenic-Like Effects of Silymarine

**Published:** 2012

**Authors:** Parichehr Yaghmaei, Shahrbanoo Oryan, Khadijeh Mohammadi, Jalal Solati

**Affiliations:** a *Department of Animal Biology, Science and Research Branch, Islamic Azad University, Tehran, Iran.*; b *Department of Biology Karaj Branch, Islamic Azad University, Karaj, Iran.*; c *Department of Biology, Karaj branch, Islamic Azad University, Karaj, Iran. *

**Keywords:** Depression, Silymarine, Serotonin, 5HT1A, Rat

## Abstract

In the present study, we have investigated the effects of silymarine on depression and the possible role of serotonergic system in these effects. The rats were anesthetized intraperitoneally with ketamine hydrochloride and placed in a Stoelting stereotaxic instrument. A stainless steel guide cannula (22-gauge) was implanted in the third ventricular region. The third ventricular region was infused by means of an internal cannula (27-gauge), terminated 1 mm below the tip of the guide cannula. Forced swimming test was used for evaluating the depression. The results obtained from this study showed that oral administration of silymarin (35, 70, 140 and 280 mg/rat) for two weeks increased the immobility time in forced swimming test, indicating an increase in depression level of the treated rats. Intra*-*third-ventricle (Intra-TV) infusion of 5HT1A receptor agonist 8-OH-DPAT (25 and 10 ng/rat) decreased the immobility time indicating an anti-depression effect, while injection of 5HT1A receptor antagonist NAN190 (0.25, 0.5 and 1 μg/rat) had no significant effect on immobility time. An effective dose of 8-OH-DPAT (10 ng/rat) co-administered with silymarin (140 and 280 mg/rat) decreased the depressogenic effects of silymarin. These results showed that the depressogenic effects of silymarin may be modulated via 5HT1A receptor of serotonin.

## Introduction

Depression is a common and pernicious illness, with a lifetime incidence. Between 15% and 20% of the patients have symptoms that persist for at least 2 years, and often do not fully recover between depressive episodes (1, 2). Depression is also associated with high rates of relapse, recurrence, disability, and death (2, 3). The relatively high rates of chronicity, relapse, recurrence, morbidity, and mortality among patients with depression underline the importance of safe and effective long-term pharmacologic treatment. However, overwhelming evidence indicates that individuals with depression are seriously undertreated and receive inappropriate or inadequate treatment, which causes enormous costs to individuals and society (4-6). 

There is good reason to believe that the use of complementary and alternative therapies is more common among people with psychiatric problems than the rest of the population since anxiety, depression and insomnia are among the most commonly reported reasons for the use of complementary and alternative therapies in community surveys (7, 8).

The results from a few previously reported studies on the use of complementary and alternative therapies among psychiatric outpatients are consistent with this speculation in showing high rates of complementary and alternative therapy usage. Consistent with this evidence, a recent national survey of using complementary and alternative therapies reported that the use of such treatments is more common among people with self-defined anxiety and depression than people with any other common chronic condition except back or neck problems (7, 9).

Milk thistle extracts have been used as traditional herbal remedies for almost 2000 years. The extracts are still widely used to protect the liver against toxins and control chronic liver diseases (10, 11).

The Milk thistle {*Silybum marianum *(L.)} is a flowering plant from the Silybum Adans genus, daisy family (Asteraceae), which grows mainly in North Africa, the mediterranean region and the Middle East. Silymarin is a polyphenolic flavonoid derived from milk thistle. It consists of three phytochemicals: silybin, silidianin and silicristin (11-15). 

Studies have shown that silymarin interacts with some neurotransmitter systems in brain and may have marginal serotonergic, dopaminergic, and noradrenergic effects (15). Silibinin, the most active moiety of silymarin, has been shown to attenuate the decrease in serotonin content of hippocampus induced by methamphetamine, and it was suggested that inhibition of the loss of serotonin could be involved in protective effect of silibinin on methamphetamine-induced cognitive impairment (15). 

The decreased levels of serotonin metabolites in cerebrospinal fluid (CSF), coupled with the mood-lowering effects of tryptophan depletion and the efficacy of serotonin-modulating antidepressants, have lent support to the notion that a dysfunctional serotonergic system is a vulnerability factor for major depressive disorder (16).

5HT1A receptor is one of the best characterized serotonin receptors, a key mediator of serotonergic signaling in central nervous system, and is involved in modulation of depression (17). 

Although sylimarin is well known for its antioxidant effects, recent studies revealed that this flavonoid interacts with central neurotransmitters such as those in serotonergic system, and may have a role in modulation of mood disorders like anxiety and depression (15). This study aimed at evaluation of the effects of silymarin on depression in rats and the possible interaction between silymarin and 5HT1A-serotonergic system in depression.

## Experimental

*Animals*

Male wistar rats from Pasteur Institute (Tehran), weighing 180-230 g at the time of surgery, were used for this study. The animals were housed four per cage in a room with a 12:12 h light/dark cycle (lights on 07:00 h) at a controlled temperature (23 ± 1°C), and they had access to food and water ad libitum and were allowed to adapt to the laboratory conditions for at least one week before surgery. The rats were handled about 3 min each day prior to behavioral testing. All the experiments were performed between 12:00 and 15:00, and each rat was tested only once. Seven animals were used in each experiment.

*Stereotaxic surgery and microinjections*

The rats were anesthetized intraperitoneally with ketamine hydrochloride (50 mg/kg) and xylazine (4 mg/kg), and placed in a Stoelting stereotaxic instrument. A stainless steel guide cannula (22-gauge) was implanted in the *third ventricular *region in accordance to the method of Paxinos and Watson (16). Stereotaxic coordinates for the *third ventricular *regions were: AP = 2.2, ML = 0 and V = -6.5. The cannula was fixed to the skull with acrylic dental cement. The animals were allowed 5 days before the test to recover from the surgery. The *third ventricle *was infused by means of an internal cannula (27-gauge) terminated 1 mm below the tip of the guides, and connected by polyethylene tubing to a 1 μL Hamilton syringe. On each side, 0.5 μL solution was injected (1 μL/rat) over a 60 sec period. The inner cannula was left in place for an additional 60 sec to allow diffusion of the solution and to reduce the possibility of reflux. Intra-third ventricular (Intra-TV) injections were made 5 min before testing.

*Forced swim test*

Forced swim test was performed as previously described (19). Briefly, the rats experienced a pre-test session followed by a test session 24 hours later. For both sessions conducted under low illumination (12 lx), the rats were placed in a plastic cylindrical tank (50 cm high by 20 cm in diameter) filled with water at 22° ± 2°C and a depth of 40 cm in which the hind limbs could not reach the tank floor. In all experiments, the pre-test was carried out for 15 min and the test for 6 min in the same tank. Following either pre-test or test sessions, the rats were dried with a towel and kept warm for 30 min before returning to their home cage. Behavioral data were collected in a blind mode by an observer quietly sitting 1 meter behind the test apparatus.

*Drug treatments*

The drugs used in the present study were Silymarin (Goldarou co. Iran), 8-oh-DPTA, and NAN190 (Sigma Chemical Co., USA). 

*Effects of silymarin on depression-like behavior*

For evaluating the effects of silymarine on depression, the rats were divided into five groups, four of which were orally given (gavage) silymarin (35, 70, 140 and 280 mg/kg; dissolved in 1 mL saline) and the fifth group saline (1 mL) for two weeks.

*Effects of 5HT1A receptor agonists and antagonists on depression-like behavior*

Three groups of rats got Intra-TV injection of selective 5HT1A receptor agonist 8-oh-DPAT (5, 10 and 25 ng/rat), and three other the 5HT1A antagonist NAN190 (0.25, 0.5 and 1 μg/rat intra-TV). These rats were thereafter compared with a saline control group.

*Effects of silymarine in combination with 5HT1A receptor agonist on depression behavior*

First, three groups of rats were orally treated with saline (1 mL/rat) or silymarine (140 and 280 μg/rat; dissolved in 1 mL saline) for two weeks. After two weeks, all groups received saline (1 μL/rat) intra-TV.

Three other groups of rats got saline (1 mL/rat) or silymarine (140 and 280 μg/rat dissolved in 1 mL saline) orally for two weeks. After two weeks, the saline and silymarine treated rats were injected 8-oh-DPAT (10 ng/rat, Intra-TV).

The behavioral tests were carried out 5 min after the Intra-TV injection of saline or drugs.

*Statistical analysis*

Since data displayed normal distribution and homogeneity of variance, one-way ANOVA was used for comparing the effects of different doses of drugs with vehicle. Two-way ANOVA was used for evaluation of the interactions between drugs. In case the difference was significant, the post-hoc analysis (least significant difference, LSD) was performed to assess the specific group comparisons. Differences between the experimental groups at each point with p < 0.05 were considered statistically significant.

## Results

*Effects of silymarine on depression behavior*

**Figure 1:**
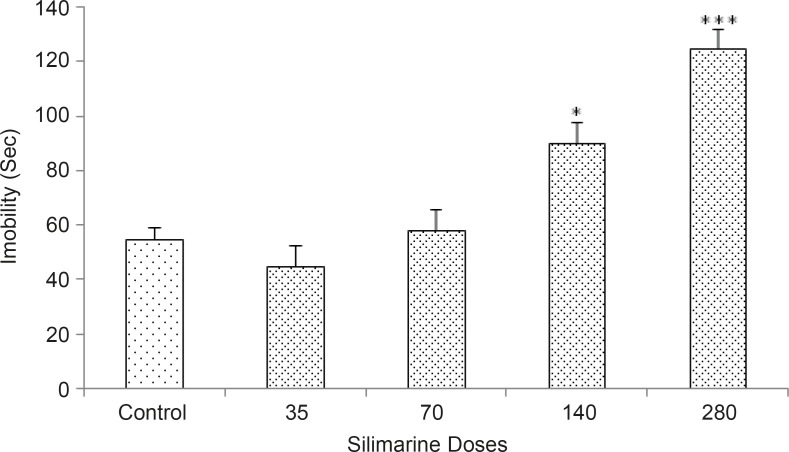
Effects of oral administration of silymarin in the forced swimming test. Rats were treated with either saline (1 mL/rat) or with silymarin (35, 70, 140, and 280 mg/rat). Each bar is mean ± SEM. n = 7. *p < 0.05 and ***p < 0.001, when compared to the saline treated rats

Figure 1 shows the effects of continued oral treatment of silymarine (35, 70, 140 and 280 mg/Kg) on depression related behaviors of the rats. The one-way ANOVA revealed that silymarine increased the immobility and % OAE [p < 0.05] at doses of 140 and 280 mg/kg, indicating the induction of increased depression response by silymarine.

*Effects of 5HT1A receptor agonist and antagonist on depression behavior*

**Figure 2:**
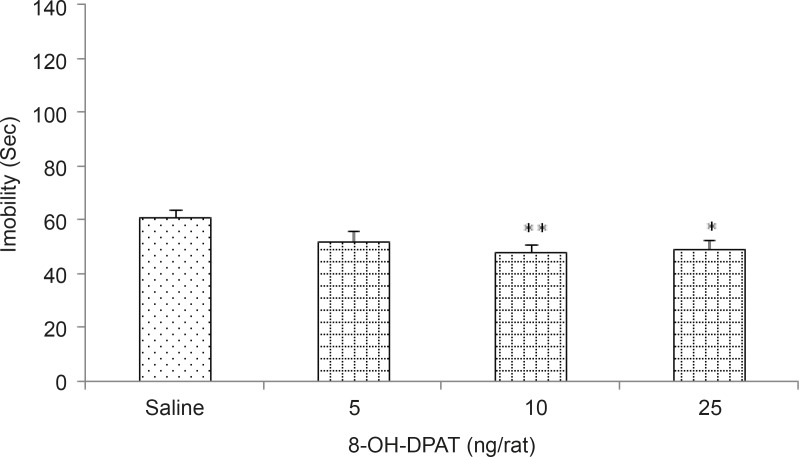
Effects of bilateral intra-third ventricle injection of 8-oh-DPAT in the forced swimming test. Rats were treated with either saline (1 μL/rat) or with 8-oh-DPAT (5, 10 and 25 ng/rat). Each bar is mean ± SEM. n=7. *p < 0.05 and **p < 0.01, when compared to the saline treated rats

Figure 2 shows the effects of intra-TV injection of the 5HT1A receptor agonist 8-oh-DPAT (5, 10 and 25 ng/rat) in the Forced swim test in rats. Our results showed that 8-oh-DPAT decreased the immobility time (p < 0.05 and p < 0.001 for the different doses respectively), indicating the induction of anti-depression response by 8-oh-DPAT. 

**Figure 3:**
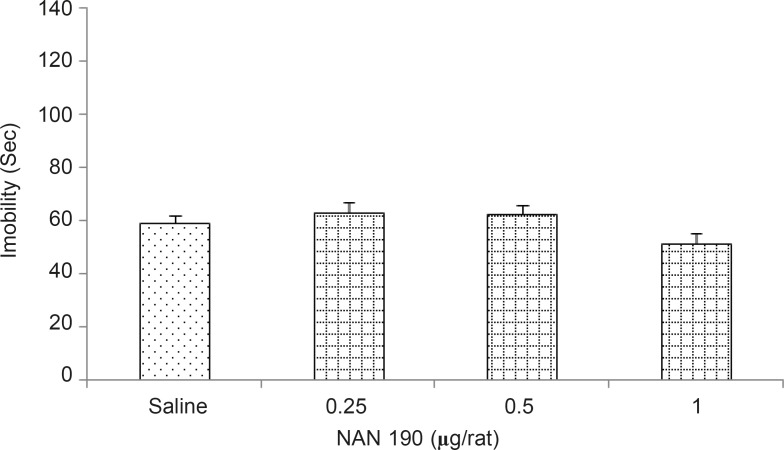
Effects of bilateral intra-third ventricle injection of NAN190 in the forced swimming test. Rats were treated with either saline (1 μL/rat) or with NAN190 (0.25, 0.5 and 1 μg/rat intra-TV). Each bar is mean ± SEM. n=7. *p < 0.05 and **p < 0.01, when compared to the saline treated rats

However, no change in immobility time was observed in rats infused with Non190 (0.25, 0.5, and 1 μg/rat, intra-TV) ( Figure 3).

*Effects of silymarine alone or with 8-oh-DPAT on depression behavior*

**Figure 4:**
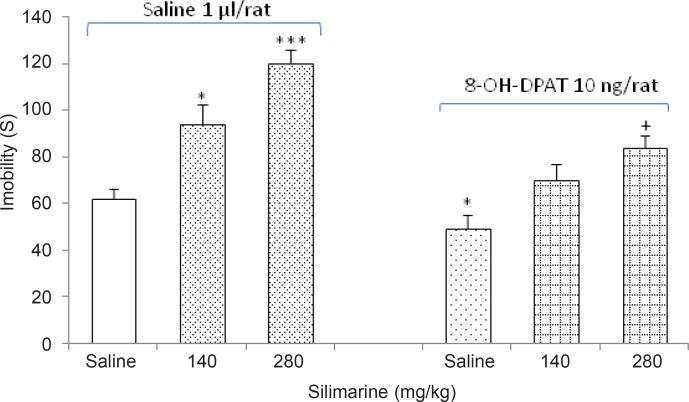
Effects of silymarin administration in the absence or presence of 8-oh-DPAT on depression-like behavior in the forced swimming test. *p < 0.05 and ***p < 0.001, compared saline/saline control group. +p < 0.05 compared to saline/8-oh-DPAT control group

The effects of intra-TV injections of the 5HT1A receptor agonist 8-oh-DPAT (10 μg/rat) on the response induced by silymarine (140 and 280 mg/Kg) are shown in Figure 4.

The two-way ANOVA indicated that the combination of silymarine (Factor A) with 8-oh-DPAT (Factor B) showed an interaction for the immobility time response [Factor A; *F *(3, 48) = 7.304, p < 0.05, Factor B; *F *(1, 48) = 8.703, p < 0.05, Factor (A × B); *F *(3,48) = 6.249, p < 0.05].

The usage rate of complementary and alternative medicine in all forms, from herbal remedies to non-medicinal therapies, has increased over the last decade from 34% to 42% of the overall population. A variety of studies suggest that this rate is higher in emotionally distressed individuals with symptoms or diagnoses of anxiety and depression. The usage rate in community has been shown to be twice as great in individuals reporting depression and anxiety than in those reporting any other problem. Scientific studies showed that some of the herbal remedies not only are ineffective but also have serious side effects (20-22). 

In the present study we have investigated the effects of silymarine, a flavonoid derived from milk thistle and one of the most frequently used herbal remedies, on depression-related behaviors of the adult male wistar rats.

Our results indicated that oral administration of silymarin at high doses (140 and 280 mg/kg) for two weeks, increased the immobility time in forced swimming test, while low doses had no significant effect on immobility time. Therefore, continued administration of silymarin at high doses leads to increased depression-like behaviors in animals. Other parts of these experiments revealed that the intra-TA injection of 5HT1A receptor agonist 8-oh-DPAT, decreased the immobility time indicating the induction of an anti-depression response by 8-oh-DPAT. Furthermore, intra-TA administration of 5HT1A receptor antagonist NAN 19 had no significant effect on immobility time. 

The results also showed that the administration of 8-oh-DPAT after two week treatment with silymarin declines the depressogenic effects of silymarin, which indicates an interaction between silymarine and 5HT1A serotonergic system. However, administration of NAN190 after continued treatment with silymarin, does not affect the responses produced by silymarin. 

According to the results, it is suggested that silymarin performs its depressogenic action via interacting with 5HT1A receptors of serotonin. 

Numerous observations support the notion that serotonin and its multiple receptor subtypes are linked not only to the biological basis of affective disorders, but also to the mechanism of action of antidepressants. Deficits in function of the 5-HT receptors have long been supposed to be implicated in many psychiatric disorders such as depression (23-25).

Over the last decade, growing evidence has suggested that the 5-HT1A receptor ligands represent a new class of antidepressant drugs. Evidence also indicates the role of 5-HT1A receptors in the mechanism of action of antidepressants (23, 25, 26). On the other hand, dysfunction of the serotonin 1A receptor (5-HT1A) may play a role in pathogenesis of major depressive disorder (16).

Basic and clinical research has shown that the sensitivity of 5-HT1A receptor may be reduced in depression. The general hypothesis of 5-HT receptor dysfunction in depression suggests that the 5-HT1A receptors may be down-regulated and show inadequate ability to convert the receptor occupancy by 5-HT into an adequate physiological response (23-27).

## Conclusions

Our results showed that the continued oral treatment with silymarine produces a depressogenic effect, while intra-third ventricular administration of the serotonin 5HT1A receptor agonist 8-oh-DPAT, decreases depression in rats. Moreover, there was an interaction between silymarin and the 5HT1A receptor agonist 8-oh-DPAT. The data possibly indicate that the depressogenic effects of silymarin are modulated at least partially by 5HT1A receptors. 
